# Direct Quantification of Natural Moisturizing Factors in Stratum Corneum using Direct Analysis in Real Time Mass Spectrometry with Inkjet-Printing Technique

**DOI:** 10.1038/s41598-019-54454-x

**Published:** 2019-11-28

**Authors:** Katsuyuki Maeno

**Affiliations:** Shiseido Global Innovation Center, 1-2-11, Takashima, Nishi-ku, Yokohama-shi, Kanagawa 220-0011 Japan

**Keywords:** Bioanalytical chemistry, Mass spectrometry, Mass spectrometry

## Abstract

Proper hydration of the stratum corneum, the skin’s outermost layer, is essential for healthy skin. Water-soluble substances called natural moisturizing factors (NMF) are responsible for maintaining adequate moisture in the skin and are closely associated with a variety of the skin’s functions. Therefore, quantitative analysis methods for NMF are indispensable when attempting to clarify one of the mechanisms of hydration and its effect on the skin. This study sought to develop a quick and simple analytical technique, which can quantify NMF from the skin without the need for extraction or separation, using direct analysis in real time-mass spectrometry (DART-MS). The goal was to deliver a high quantitative capability, so a unique inkjet printing technique was employed to evenly coat a stable isotope-labeled internal standard (SIL-IS) on tape-stripped skin. This technique allowed for the quantification of 26 NMF with established calibration curves and comparatively high linear correlations. The speed of measurement was found to be advantageous as 100 strips of tape can be measured in roughly 2 hours. The effectiveness of the inkjet coating was also verified by comparing its precision with that of conventional pipetting. This new technique can be an alternative method to quantify NMF rapidly and perhaps allow for a clearer elucidation of their function in skin.

## Introduction

Ambient mass spectrometry (MS), such as direct analysis in real time (DART)^1,2^, desorption electro spray ionization (DESI)^3,4^, low temperature plasma (LTP)^5^, and paperspray^6^, is an ambient ionization method that reduces the time, equipment, and expertise needed for sample preparation and chromatography separation. There are portable applications with ambient MS because measurement procedures are simple and quick to execute^7–10^. Solid or liquid samples can be measured simply by placing them in the gap between the ion source and the inlet of the mass spectrometer. DART-MS is considered an effective ambient plasma ionization method that can dramatically reduce analysis time for the routine screening of samples. Therefore, DART-MS has been successfully applied in a variety of fields such as foods^11^, food packaging^12^, forensic analysis^13^, additives in plastics^14^, contaminants in soil^15^, pesticides^16^, metabolites^17,18^, drugs^19^, nucleotides^20^, and mycotoxins^21^. The DART ion source can supply a stream of electrically discharged gas (typically excited He or N_2_) and samples are hit directly by those gases or indirectly via other reactions with water molecules to form the ionized components that lead to the MS^1,2^. Since the time of the DART method’s development, a lot of qualitative applications have arisen by combining DART with high resolution MS^22–24^ and ion mobility^25^. While quantitative applications were initially few, they are now increasing through the use of stable-isotope-labeled (SIL) internal standards (SIL-IS) and other techniques^22,26–30^.

We developed a method to quantitatively analyze natural moisturizing factors (NMF) using DART-MS and SIL-IS in our previous study^31^. NMF, such as urea, pyrrolidone carboxylic acid, lactic acid, urocanic acid, and various amino acids, are water-soluble compounds with low molecular weights that exist in the epidermis^32,33^. The epidermis, the upper layer of skin, has the ability to produce NMF and they are responsible for maintaining adequate hydration of the stratum corneum (SC), the outermost part of the epidermis^34–37^. Proper hydration is essential for healthy skin in terms of elasticity, enzyme activity, and barrier function^38–42^. In addition, NMF are involved in some diseases, such as ichthyosis and atopic dermatitis^43,44^. Therefore, the mechanisms of NMF production and the relevant enzymes associated with them have become a prominent area of focus and study. In measuring NMF, SC samples are collected simply by tape-stripping and are quantified using liquid chromatography-mass spectrometry (LC-MS) or gas chromatography-mass spectrometry (GC-MS). Tape-stripping is a technique to take a SC sample using adhesive tape. The procedure is as follows. A tape is firmly adhered to the skin, pressed with the fingers over the entire area covered by the tape, and then removed from the skin. While a large quantity of sample skin is obtainable thanks to this simple method, the chromatographic techniques require lengthy analyses and cannot meet current levels of demand. In order to overcome this issue, we used DART coupled with time-of-flight (TOF) MS and were able to quantify 12 NMF in the previous report^31^.

However, there were still 2 issues to be tackled. One was that only 12 NMF among 26 were quantified because some components were too few to detect in the samples. In addition, leucine and isoleucine were not separable without chromatographic techniques. The second was the lower accuracy resulting from the addition of SIL-IS. Ambient mass spectrometry methods, such as DART-MS, are more susceptible to ion suppression/enhancement^45,46^, and the tissue-specific ion suppression effect still provides challenges for quantitative analysis^47^. Although SIL-IS are thought to be essential for quantitative analysis, they are difficult to coat evenly as the SC samples are solid.

We employed a triple quadrupole (TQ) MS suitable for quantitative analysis, owing to its high sensitivity using multiple reaction monitoring (MRM). NMF that are undetectable using TOF MS can possibly be detected using TQ. In addition, more selective identification can be achieved using the product ions formed via the collisions. For example, leucine and isoleucine have the same molecular composition. Thus, they cannot be distinguished without chromatographic separation. However, the product ions induced by collision-induced dissociation are different. They can be separated in an identical fashion using TQ. Furthermore, an inkjet printing technique was used to realize homogeneous deposition and coating of the SC samples to improve the accuracy. This technique has been already utilized in the field of imaging MS (IMS) for matrix additions^48^, calibration standards^49^, and SIL-IS^50^. However, our inkjet technique is unique and superior in terms of both the precision and volume of ejected droplets, allowing for highly customized coating patterns.

Described below are the steps to be taken to establish the quantification method for NMF in the SC using the inkjet printing technique and TQMS. Introduced is a novel procedure for high-throughput and quantitative analysis using DART-MS.

## Results and Discussion

### Workflow of the NMF quantification

**Figure 1:**
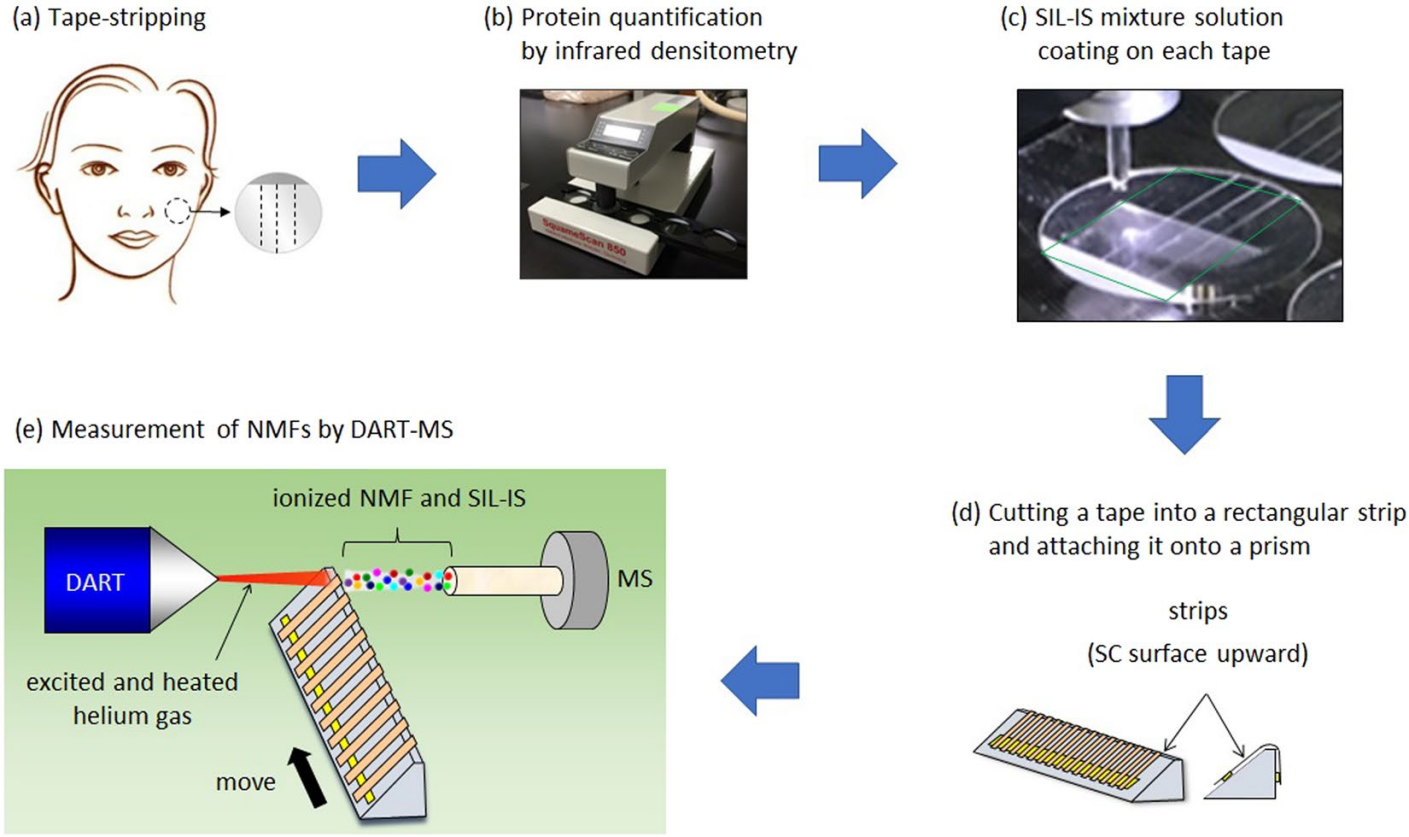
Workflow of the NMF measurement. (**a**) SC sample is taken from skin with an adhesive tape. (**b**) Amount of total proteins in the SC adhered to the tape is quantified by infrared densitometry and used to normalize the value of NMF. More specifically, the amount of NMF quantified by DART-MS is divided by the amount of proteins, which is considered to be proportional to the amount of SC. (**c**) The SIL-IS mixture solution is coated on each tape with LaboJet. The SIL-IS mixture solution is coated inside the green square. (**d**) A tape is cut into a rectangular strip and attached onto a prism with a double-faced adhesive tape. 10 tapes are placed on the same prism. (**e**) The prepared prism is set between a DART ion source and a MS detector so that excited and heated helium gas hits the strips. NMF and SIL-IS are evaporated and ionized and go into the MS detector.

The goal of this study was to establish and optimize a procedure for NMF quantification in the SC which is faster and simpler than conventional techniques and which includes all the steps needed from sample collection to data analysis. The established workflow is shown in Fig. 1. First, SC samples were collected from various parts of the body from volunteers with tape stripping using D-squame tape (Cuderm Corporation, Dallas, TX, USA) with a diameter of 22 mm (Fig. 1a). The tape was originally slitted so that the tapes could easily be cut into rectangular strips for DART-MS measurement later. The tape stripping was conducted several times on the same area of skin to examine the depth profile of NMF. Then, the amount of total proteins in the SC adhered to the tape was measured using a SquameScan 850 A instrument (Heiland electronic, Wetzlar, Germany, Fig. 1b)^51^. Since the amount of SC taken from the skin varies, it is not possible to attribute the amount of NMF to the quality of SC or the amount of SC. Knowing the weight of SC removed is beneficial to distinguish those 2 factors, and we used the amount of total proteins as a key indicator in this study. Since the amount of total proteins measured is considered to be proportional to the amount of SC removed, that value was used to normalize the amount of NMF measured using DART-MS^21^. After that, the tape was placed in the inkjet device and coated with a SIL-IS mixture solution (Fig. 1c). A square shape area with one side of 11 mm (a green square area in the tape in Fig. 1c) was coated so that the area used for the DART-MS measurement could be included. One nL of the SIL-IS mixture solution was ejected from the inkjet head and dropped on the surface of the tape containing the SC. The droplets were designed to lie in a laterally and longitudinally aligned grid pattern of spots with a distance of 220 μm between each droplet (Fig. S1). Each droplet quickly evaporated and dried. The final concentration of the coated SIL-IS was 1 nmol/cm^2^ on the tape for each SIL-IS. 81 tapes (9 × 9) can be accommodated on the stage part of this device and coated within 1 hour at one time. Then, the tape was cut into 2 rectangular strips (20 × 2 mm) (Fig. 1d). The strip was attached to the top of a quartz prism (Fujiwara, Tokyo, Japan) with the adhesive side facing up. 10 strips were attached onto the same prism at intervals of about 10 mm between each strip. The prism was placed between the DART ion source and the MS detector so that the excited and heated helium gas from DART hit the surface of the tape strips on the prism. The helium gas reacts with atmospheric water molecules to produce ionized water clusters [(H_2_O)_n_ + H]^+^. These protonated water clusters can then react with the NMF and SIL-IS to form protonated cations and then flowed into the ceramic tube inlet for MS analysis (Fig. 1e). Heated helium is needed to evaporate NMF in the SC and SIL-IS coated on the SC adhere to the strips before ionization. The prism moved automatically at a speed of 0.2 mm/s in a direction perpendicular to the flow of helium gas. We chose a distance of 10 mm between 2 tapes and set the speed of the prism at 0.2 mm/s so that the signals of all the NMF and SIL-IS were consumed before the peak of the next tape appeared. The area hit by the helium gas was approximately 0.1 cm^2^ (10 × 2 mm), which was measured using heat-sensing tapes (NiGK, Saitama, Japan) placed on the quartz prism instead of the D-squame tape. Fig. S2 shows a typical total ion current chromatogram for one prism. MRM chromatograms measured with optimized MRM methods for each NMF were produced, and the peak area was used to determine the amount of NMF. 10 tapes are measured at one time within about 10 minutes.

### MRM method selection for NMF and SIL-IS

A number of recent studies have shown that DART forms abundant (de)protonated analytes [M ± H]^±^ via a proton transfer from background ions such as H_3_O^+^(H_2_O)_n_ and O_2_ to the analytes M with relatively low internal energy^29,52^. That is also applicable to NMF. Another group has confirmed that almost all amino acids produce (de)protonated molecules at the dominant ion peaks^52^. Our study also obtained the same result. Therefore, (de)protonated molecules of NMF and SIL-IS were used as precursor ions for the MRM conditions. However, specific MRM methods for NMF and SIL-IS must be applied because chromatographic separation is not possible and they are only distinguishable using MS. It is probable that a method optimized for a specific NMF or SIL-IS will detect others because most NMF and SIL-IS have a carboxylic group and an amino group in common and their molecular composition or structures, such as aspartic acid and asparagine, glutamic acid and glutamine, are similar to each other. We discovered about 10 optimized MRM conditions for each NMF and SIL-IS through an MRM optimization tool installed in LCMS8040 and confirmed the selectivity of each MRM method as follows. A solution containing 100 ppm of each of the NMF and SIL-IS was measured under the MRM conditions optimized for each NMF and SIL-IS by placing a capillary tube with a small amount of each solution between the DART ion source and the MS. We managed to find specific MRM conditions for almost all NMF and SIL-IS except for leucine and isoleucine, as shown in Table 1. Leucine and isoleucine are structural isomers that share the same chemical formula. Therefore, both molecules produce similar product ions and are difficult to distinguish without chromatographic separation. Fig. S3 shows the specificity result of leucine and isoleucine with the best MRM methods optimized for each of the 2. The MRM method optimized for isoleucine (*m/z* 132.1 > *m/z* 57.1) detects isoleucine by the product ion *m/z* 57.1 originating from the branched structure. However, it also detects leucine with a relatively small peak compared to isoleucine. Leucine and isoleucine are considered to be present in SC in the same representative amounts, as shown in the GC-MS result, so we decided to use this method for isoleucine. On the other hand, the MRM method optimized for leucine (*m/z* 132.1 > *m/z* 43.1) detects only leucine by the product ion *m/z* 43.1. In addition, some NMF, such as cysteine, glycine, lactic acid, urea, alanine, do not give enough product ions to be detectable. Therefore, selected ion monitoring (SIM) for (de)protonated ions was used instead of MRM. In this study, we employed some SIL-IS with only one isotope label because of the low price. However, a mass of a naturally occurring isotope in the NMF to be measured can overlap the mass of its SIL-IS. Therefore, theoretical isotope ratios of NMF were calculated and the amounts of NMF affecting the amounts of SIL-IS were taken into consideration to make the calibration curves.

**Table 1:** Summary of method validation parameters for 26 NMF.

NMF	MRM or SIM condition		SIL-IS	MRM or SIM condition		Dynamic range [nmol/cm^2^]	Slope ± SD	Intercept ± SD	R^2^	%RSD									LOQ [nmol/cm^2^]
	Transition	EV		Transition	EV					NMF conc. [nmol/cm^2^]	25	5	2.5	1.25	0.5	0.25	0.1	0.05	
Val	118.10 > 72.20 (+)	-12	ValSIL	119.10 > 73.15 (+)	−11	0.05–25	7.9 ± 0.34	3.9 ± 0.59	0.998		4	5	9	7	13	10	2	14	0.23
Asn	131.05 > 113.10 (−)	14	AsnSIL	133.10 > 115.10 (−)	16	0.05–5	8.0 ± 0.21	−0.39 ± 0.039	0.979		—	16	14	4	2	11	6	10	0.60
Asp	132.05 > 115.05 (−)	15	AspSIL	137.00 > 92.05 (−)	13	0.05–5	1.4 ± 0.1	0.43 ± 0.074	0.964		—	7	12	3	6	11	10	5	0.57
Cit	174.10 > 131.10 (−)	13	AsnSIL	133.10 > 115.10 (−)	16	0.05–2.5	0.69 ± 0.042	−0.013 ± 0.023	0.991		—	—	15	14	11	3	7	5	0.86
Cys	122.05 (+)	—	MetSIL	151.10 > 61.05 (+)	−22	0.5–5	1.8 ± 0.26	0.93 ± 0.15	0.958		—	15	31	9	8	—	—	—	2.6
Gln	147.10 > 84.10 (+)	−18	AsnSIL	135.10 > 75.10 (+)	−15	0.05–5	43 ± 1.5	0.2 ± 2.5	0.987		—	5	11	14	5	13	10	20	0.38
Glu	146.05 > 102.10 (−)	15	GluSIL	147.10 > 103.10 (+)	−15	0.25–5	1.8 ± 0.26	2.0 ± 0.31	0.833		—	13	15	13	11	15	—	—	1.2
Gly	76.1 (+)	—	AlaSIL	91.10 > 45.10 (+)	−15	0.5–5	3.5 ± 0.67	−1.3 ± 0.31	0.974		—	19	12	4	15	—	—	—	3.7
His	156.10 > 110.05 (+)	−15	HisSIL	159.10 > 113.10 (+)	−15	0.05–25	3.4 ± 0.29	3.3 ± 0.74	0.99		8	11	17	14	18	8	19	6	0.34
Ile	132.10 > 69.10 (+)	−16	LeuSIL	133.10 > 87.10 (+)	−11	0.05–25	1.0 ± 0.19	−0.15 ± 0.28	0.998		18	4	8	6	17	16	10	8	0.46
Leu	132.10 > 43.10 (+)	−26	LeuSIL	133.10 > 87.10 (+)	−12	0.05–25	0.5 ± 0.063	0.24 ± 0.11	0.999		12	14	25	11	10	10	3	22	0.20
Lys	147.10 > 67.15 (+)	−25	LysSIL	149.10 > 57.15 (+)	−25	0.05–5	5.6 ± 0.69	0.33 ± 0.53	0.938		—	13	34	5	6	13	11	11	0.43
Met	150.05 > 56.15 (+)	−17	MetSIL	151.10 > 61.05 (+)	−22	0.05–5	8.7 ± 0.33	−0.016 ± 0.66	0.993		—	6	14	12	4	9	24	12	0.37
Orn	133.10 > 70.15 (+)	−17	LysSIL	149.10 > 67.15 (+)	−25	0.05–2.5	120 ± 11	9.7 ± 5.3	0.928		—	—	7	3	9	8	8	10	0.70
PCA	128.10 > 84.05 (−)	14	PCASIL	133.10 > 89.10 (−)	15	0.05–5	1.7 ± 0.12	0.64 ± 0.11	0.978		—	7	10	8	4	18	6	3	0.12
Phe	166.10 > 120.05 (+)	−15	PheSIL	171.10 > 125.15 (+)	−15	0.05–5	8.9 ± 0.1	1.4 ± 0.2	0.992		—	2	4	7	9	7	8	13	0.073
Pro	116.05 > 70.10 (+)	−16	ProSIL	117.10 > 71.10 (+)	−16	0.05–25	5.1 ± 0.41	−0.16 ± 0.83	0.999		8	22	42	13	7	6	14	17	0.22
Ser	106.05 > 60.05 (+)	−11	SerSIL	105.10 > 74.10 (−)	15	0.05–25	31 ± 1.7	−0.033 ± 0.39	0.899		21	12	18	5	15	3	19	4	0.15
Thr	120.05 > 56.00 (+)	−15	AsnSIL	133.10 > 115.10 (−)	16	0.05–5	4.0 ± 0.21	2.2 ± 0.23	0.896		—	5	8	11	6	6	5	11	0.14
Trp	205.10 > 188.10 (+)	−11	TrpSIL	207.10 > 189.00 (+)	−10	0.05–5	2.9 ± 0.51	0.82 ± 0.51	0.835		—	16	56	12	18	14	12	8	0.37
Tyr	182.10 > 91.10 (+)	−28	TyrSIL	183.10 > 137.15 (+)	−13	0.05–5	8.8 ± 1.3	0.43 ± 1	0.993		—	16	35	4	8	18	8	14	0.37
UCA	137.10 > 93.05 (−)	14	AspSIL	137.00 > 92.05 (−)	13	0.05–5	4.8 ± 0.43	2.1 ± 0.17	0.921		—	8	10	8	8	5	9	6	0.32
LA	89.1 (−)	—	LASIL	92.1 (−)	—	0.5–5	1.6 ± 0.26	0.96 ± 0.18	0.974		—	15	24	5	5	—	—	—	3.9
Urea	61.0 (+)	—	UreaSIL	62.0 (+)	—	0.25–5	0.39 ± 0.038	0.13 ± 0.024	0.948		—	10	14	11	10	6	—	—	1.2
Arg	175.10 > 70.10 (+)	−17	LysSIL	149.10 > 57.15 (+)	−25	0.25–5	0.51 ± 0.035	−0.084 ± 0.18	0.97		—	7	11	15	12	18	—	—	1.7
Ala	88.0 (−)	—	AlaSIL	91.10 > 45.10 (+)	−15	0.05–5	2.3 ± 0.21	0.38 ± 0.8	0.969		—	9	16	9	6	8	10	15	0.34

The actual concentration values used are 0.05, 0.1, 0.25, 0.5, 1.25, 2.5, 5, 25 nmol/cm^2^. Amino acids are described with three-letter abbreviations. Cit, citrulline; Orn, ornithine; PCA, pyrrolidone carboxylic acid; UCA, urocanoic acid; LA, lactic acid; (+), positive ion mode; (−), negative ion mode; EV, electrical voltage; R^2^, correlation coefficient; LOQ, limit of quantification.

### Calibration curves for NMF

A summary of the calibration curves of the 26 NMF is shown in Table 1. A validation process was performed by determining the linear range, precision, and limit of quantification (LOQ) based on the procedure defined by the Food and Drug Administration (FDA). Blank tapes coated with the mixture solutions of NMF and SIL-IS by LaboJet were used to determine the validations. Matrix-specific validation is often desired owing to the presence of different interfering components. Due to the presence of unknown amounts of endogenous NMF in this study, the SC cannot be used directly as a blank. Furthermore, the SC is a heterogeneous solid sample and cannot be separated into several even portions. Therefore, different approaches, such as surrogate analytes and standard additions, could not be employed. The slopes, intercepts, and correlation coefficients are shown as characteristic parameters of linearity in the range from 0.05 to 25 nmol/cm^2^. All the NMF were detectable (S/N > 3) at 0.05 nmol/cm^2^, except for lactic acid, urea, arginine, glycine, cysteine, and glutamic acid, which were detectable at 0.25–0.5 nmol/cm^2^. The low detectability can be ascribed to their low volatility or SIM methods. Good linear correlations for all of the NMF in a given concentration range were obtained between the peak area ratio of the NMF to IS and the amount of NMF applied to each piece of tape. Repeatability (intraday) was also assessed by measuring the tape 8 times. The relative standard deviation (RSD) was calculated for all concentration levels as an indicator of the intra-assay precision and only the maximum RSD values among all of the concentrations are shown. The data indicates relatively good intraday precision. The LOQ was determined based on a signal-to-noise ratio (S/N) of 10, where the signal is the peak intensity of each NMF extracted from the chromatogram. The amount of NMF depends on the place of the body and also the amount of SC taken. Looking at the SC in the forearm and cheek, the amounts of arginine and lactic acid present were lower than their LOQ, respectively, so these must be improved in the future.

### Comparison of addition techniques of the SIL-IS mixture solution (LaboJet vs Micropipetto)

**Figure 2:**
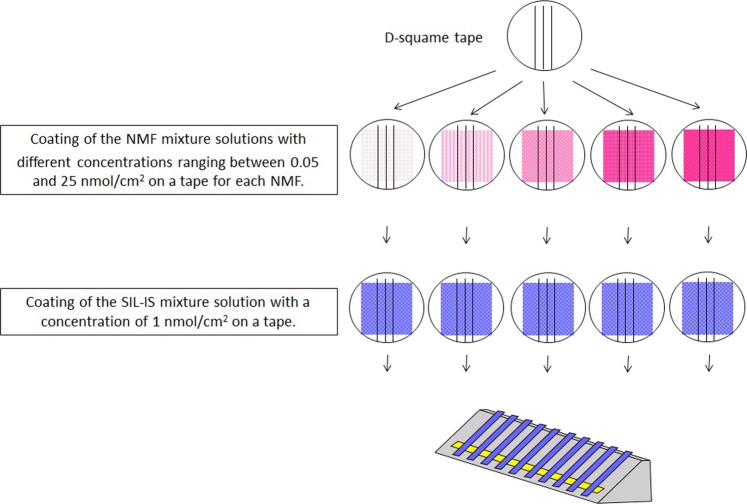
Preparation for standard tapes for calibration curves. Pink square areas of D-squame tapes are coated with the NMF mixture solutions with different concentrations. (A pink color density represents the concentration of the NMF mixture.) Then, the blue square areas are coated with the SIL-IS mixture solution with a concentration of 1 nmol/cm^2^ on a tape. After that, those tapes are set on the prism and measured by DART-MS.

**Figure 3:**
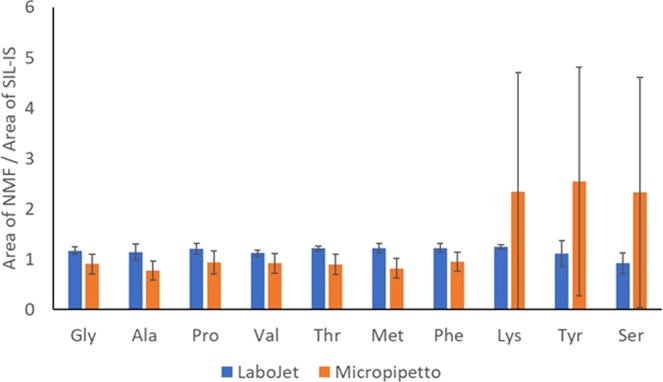
Comparison of 2 addition methods for the SIL-IS mixture solution. Tapes were measured 8 times for each method respectively.

To demonstrate how effective the LaboJet coating is, we compared the IS addition by Labojet with the Micropipetto used in the previous study^31^. D-squame tapes coated with a concentration of 1 nmol/cm^2^ on the tape for each NMF were prepared in the same way as in Fig. 2. Then, the SIL-IS mixture solution was added onto the tape using 2 different coating methods, LaboJet and Micropipetto. For the LaboJet addition, the SIL-IS mixture solution was applied onto the tape in the same way as in Fig. 2. For the Micropipetto addition, 1 μL of 100 nmol/mL of the SIL-IS mixture solution was applied within the central 2 × 5 mm area (0.1 cm^2^) of the tape, which is the area He gas hit, as shown in the previous report^31^. So, the final amounts of SIL-IS added by both methods on the tapes were the same, 1 nmol/cm^2^. After that, both tapes were set on the prism and measured by DART-MS in the same way as described in Fig. 1 6 times, respectively. %RSD for all NMF measured with the LaboJet addition technique were smaller than those with the Micropipetto addition (Fig. 3, Table S1). He gas hits an area of about 0.1 cm^2^ (2 mm × 5 mm) on the tape. For the addition by Micropipetto, it is possible that the added SIL-IS mixture solution is not evenly coated and the quantified values are likely to be higher than the actual values or fluctuate as shown in Fig. 3. In addition, the area of tape He gas hit is not necessarily constant. The DART ion source (He gas), the top of the prism, and the MS detector are designed to lie on a straight line. If the position of the DART is higher, even by a few mm, than the optimized position, the He gas passes over the tape on the prism and results in an area of tape smaller than 0.1 cm^2^ being hit by the gas. On the contrary, if the position of DART is lower than the optimized position, the He gas hits a lower position of the tape and the area getting hit becomes larger than 0.1 cm^2^. In both cases, the ratio of the amount of NMF to the amount of SIL-IS could vary because SIL-IS is not coated evenly on the tape and exists at random at certain positions in the area the He gas is hitting. Therefore, the fluctuation of the height of DART leads to a larger %RSD as well as higher quantified values. Lysine, tyrosine, and serine showed much larger %RSD. The values of the area of NMF divided by the area of SIL-IS were higher than those of other NMF. Theoretically, the ratio of the area of NMF to the area of SIL-IS should be one. That has still not been elucidated and is under investigation. However, our hypothesis is that the physical characteristics of NMF have something to do with the result (isoelectric point, volatility, polarity, etc.). On the other hand, the ratio of the amount (area) of SC to the amount of SIL-IS measured by LaboJet is always the same regardless of the position that the He gas hits. This result proved that Labojet coating is essential for a precise, direct quantification of solid samples.

### Comparison of quantitative NMF values obtained by DART-MS and GC-MS

**Figure 4:**
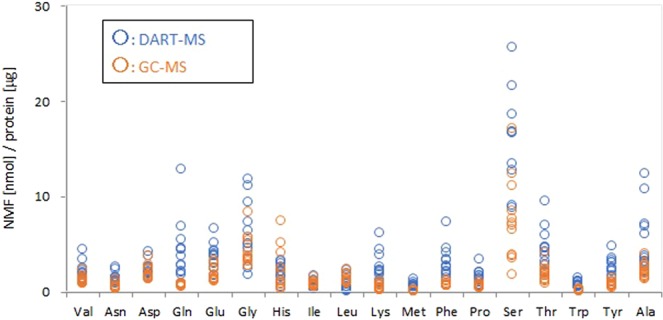
Comparison of the quantitative NMF values obtained by DART-MS and GC-MS. An individual quantitative value for each SC stripped tape obtained by DART-MS and GC-MS.

**Figure 5:**
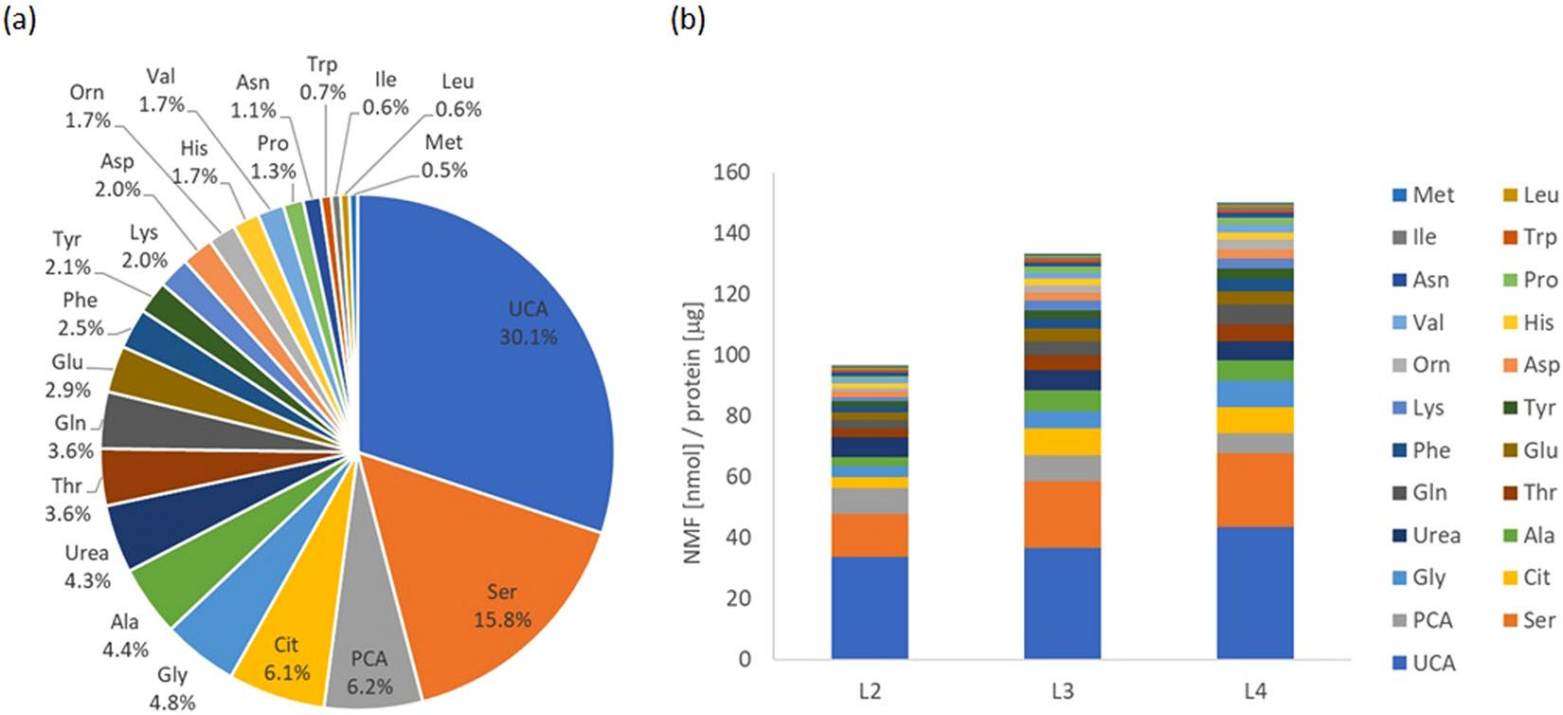
NMF profiles obtained by DART-MS. (**a**) The NMF compositional profile. Each percentage is the average of 12 tapes. (**b**) The NMF depth profile. L2 is an average NMF value of all the 2^nd^ tapes from the 4 volunteers. L3 is an average NMF value of all the 3^rd^ tapes from the 4 volunteers. L4 is an average NMF value of all the 4^th^ tapes from the 4 volunteers.

A standard addition technique is commonly used to overcome any matrix interferences occurring between the target molecules and matrix in a sample. However, this technique involves adding known amounts of standard to one or more aliquots of the processed samples. It cannot be applied to solid samples, such as the SC because the SC is a heterogeneous sample and identical samples cannot be prepared. Therefore, we employed another analytical method for the NMF quantification to verify our technique. The amounts of NMF measured by DART-MS were compared with those measured by a conventional GC-MS method. This GC-MS method has been previously validated, but it is designed to measure only amino acids^53–55^. Therefore, the amounts of amino acids obtained by the 2 methods were compared. A tape-stripped SC sample was cut in 2, with one half measured by DART-MS and the other by GC-MS, as described in Materials and Methods. These 2 areas were next to each other. Therefore, we assumed that these 2 areas contained the same levels of amino acids. Figure 4 shows the amounts of amino acids with profiling obtained by DART-MS and GC-MS. We measured 12 samples in total (the 2^nd^, 3^rd^, and 4^th^ tapes from 4 volunteers) with each method. Some amino acids showed good agreement in the values between DART-MS and GC-MS. However, the ratio of the recovery of amino acids obtained by DART-MS to the one obtained by GC-MS was not consistent. Most of the amino acids showed that the amounts obtained by DART-MS were 2 to 5 times larger than those obtained by GC-MS. One possible explanation for this difference is that amino acids are extracted from a liquid for the GC-MS, while they are simultaneously evaporated and ionized by heated and excited He gas for the DART-MS. It is possible that the latter method has a higher extraction efficiency. Another explanation for the result is that a difference in the states of NMF and SIL-IS in the SC solid sample affects the recovery obtained by DART-MS because the NMF are originally present in the SC while the SIL-IS are added on the SC and penetrate into the SC. Therefore, the positions at which NMF and SIL-IS exist cannot be the same. Basically, NMF should be in the same depth and state as SIL-IS. Further study is necessary to elucidate the difference of the recovery. An example of NMF compositional profile in the SC taken from the cheek using DART-MS is shown in Fig. 5a. All values are shown in Table S2. Among the 26 NMF, the values of arginine, cysteine, and lactic acid were lower than the LOQ and were undetectable. Therefore, the remaining 23 NMF were quantified using the calibration curves described above. The NMF compositional profile was not so different from the one which was already reported^56^. Serine, pyroglutamic acid, citrulline, glycine, alanine and urea were present in the SC as major components. An NMF depth profile in the SC was also examined (Fig. 5b). Most of the NMF showed an increasing trend with the depth of the SC. In contrast, urea, which is the NMF originating from sweat, showed a decreasing trend. Although these trends have already been shown by other studies, our DART-MS technique provided further confirmation of these trends^35,56^.

## Conclusions

A novel analytical method for the rapid and easy quantification of NMF in the SC was successfully achieved by combining the DART-MS technique and SIL-IS coating technique using LaboJet. DART-MS is a rapid and easy-to-use analytical technique. However, quantification precision is a disadvantage of DART-MS. We improved that by employing a new SIL-IS coating technique with LaboJet and proved that SIL-IS, coated evenly in a reticular pattern with nanoliter droplets, generated low %RSD of quantitative values and could be used to enhance the precision of the DART-MS quantification technique. Good linear correlations were also obtained for the calibration curves of 26 NMF with this coating technique. The developed DART-MS technique does not require pretreatment, such as liquid extraction and chromatographic separation. This result means a reduced analysis time compared to conventional techniques, with only 2 hours being required to measure 100 samples. To the best of our knowledge, other than this study, there is no report on direct quantification of NMF in the SC. At present, we are aiming to advance this DART-MS technique to on-site analysis by shortening the SIL-IS coating time. Furthermore, DART-MS is not limited to NMF analysis. It can detect other components, such as lipids, in the SC.

## Materials and Methods

### Materials and preparation

All of the amino acids (alanine, arginine, asparagine, aspartic acid, cysteine, glutamic acid, glutamine, glycine, proline, serine, tyrosine, histidine, isoleucine, leucine, lysine, methionine, phenylalanine, threonine, tryptophan, valine, ornithine, and citrulline), pyroglutamic acid, urocanic acid, urea, and lactic acid were purchased from Wako (Osaka, Japan). All of the SIL-IS were purchased from JUNSEI (Tokyo, Japan). Deionized water was produced in-house using a Milli-Q gradient water purification system (Merck Millipore, Darmstadt, Germany). The SIL-IS mixture solution containing all of the SIL compounds was prepared in deionized water with each having a final concentration of 0.5 μmol/mL. The NMF mixture solutions containing all of the amino acids and pyroglutamic acid, urocanic acid, urea, lactic acid were prepared in deionized water with a final concentration ranging between 0.025–12.5 μmol/mL for each compound.

### DART-MS

DART-MS measurements were performed using a DART ion source (IonSence, Saugus, MA, USA) and a triple quadruple mass spectrometer LCMS8040 (Shimadzu, Kyoto, Japan). The ion source was operated in positive ion mode at 500 °C. Helium was used as the ionizing gas at a flow rate of 3 L/min. A MS ion source was not used (Ionization voltage, off; Nebulizing gas, off; Drying gas, off). The desolvation line temperature was set at 250 °C. All spectra were acquired in a mass range of *m/z* 50–600. The positive/negative switching mode was used in correspondence to the components. A dwell time of all of the MRM and SIM methods for NMF and SIL-IS was 10 ms and a loop time, which is the time between data points, was 0.8 s. One peak consists of about 12 data points on average, although peak width varies depending on the molecule.

### LaboJet

The SIL-IS mixture solution and the NMF mixture solution were printed on the SC samples evenly using LaboJet2000 (MicroJet, Nagano, Japan). It has a piezoelectric system that can dispense a droplet ranging from 1 pL to 1 nL precisely. The volume of the droplet can be varied by changing the electric voltage applied to the piezoelectric dispenser. Operating conditions for the piezoelectric dispenser varied slightly day-to-day depending especially on atmospheric pressure and temperature. To demonstrate the homogeneity and stability, and therefore resulting high accuracy, of the amount ejected with LaboJet, masses of 100,000 droplets of water were determined by weighing them with an analytical microbalance each time before the coating; the mass of 100,000 droplets of water is 100 mg. We always made sure that the value was within a 1% error margin. This inkjet device not only dispenses a precise amount of fluid but places the droplet on an accurately aimed location. Therefore, droplets can be deployed with a set distance between each other in a pre-determined pattern. When coating SIL-IS, it was ensured that no droplet would overlap the other droplets around it and there were always the same number of droplets, or the same amount of SIL-IS in a certain area of the sample, which made a grid pattern of spots inside a rectangular area, as shown later. This inkjet technique was also used to make a pseudo-skin sheet to establish and evaluate this new analytical method, along with the SIL-IS coating.

### MRM method optimization for NMF and SIL-IS

MRM conditions (mainly electrical voltage [EV] values and product ions) for NMF and SIL-IS were set using an MRM optimization technique equipped with LCMS8040 by flowing each 10 ppm solution. Some components were too small to be fragmented, so a selected ion monitoring (SIM) mode was adapted instead of the MRM mode. SIL-IS are the best IS for precise quantification, where one or more atoms are replaced by heavy stable atoms, such as D, ^14^C, ^15^N, ^18^O, and so on. However, some components do not have the SIL-IS. In that case, we employed another SIL-IS which has a similar structure and characteristics to the component to be measured.

### Comparison on the quantitative NMF values obtained by DART-MS and GC-MS

To compare the data from our DART-MS technique with a conventional one, we employed a GC-MS method using EZ:faast (Phenomenex, CA, USA). EZ:faast is an easy-to-use kit that contains everything needed for sample clean up, derivatization, and analysis of amino acids by GC-MS^53^. All of the volunteers in this study were healthy Japanese adults in their 30 s. Informed consent was obtained from all of them. This human study was approved by the Shiseido Committee on Human Ethics and all methods were carried out in accordance with relevant guidelines and regulations. SC samples were obtained from the cheeks of the 4 volunteers by tape stripping with D-squame tape. Tape-stripping was performed 4 times on the same location of the cheek and the 2^nd^, 3^rd^, and 4^th^ tapes were used for measurements. Each tape was cut in 2, one (5 × 2 mm) is for DART-MS measurement and the other (10 × 5 mm) is for GC-MS measurement, which were next to each other. For the GC-MS measurement, the strip was analyzed by EZ:faast as follows: Each strip was immersed in 500 μL of 10 mmol/L HCl, and the amino acids were extracted by sonication for 5 min and incubation for 24 hours^54^. Then, the supernatant was obtained by centrifugation and analyzed by GC-MS. A GCMS-QP2010 Plus (Shimadzu, Kyoto, Japan) gas chromatograph-mass spectrometer was used to quantify the amino acids.

## Supplementary information

supplemental data

## Data Availability

Datasets generated during and/or analyzed in the current study are not publicly available but can be requested from the author.

## References

[1] Cody RB, Laramee JA, Durst HD (2005). Versatile new ion source for the analysis of materials in open air under ambient conditions. Anal. Chem..

[2] Gross J (2014). Direct analysis in real time-a critical review on DART-MS. Anal. Bioanal. Chem..

[3] Cooks RG, Ouyang Z, Takats Z, Wiseman JM (2006). Ambient mass spectrometry. Science..

[4] Takats Z, Wiseman JM, Gologan B, Cooks RG (2004). Mass spectrometry sampling under ambient conditions with desorption electrospray ionization. Science..

[5] Harper JD (2008). Low-temperature plasma probe for ambient desorption ionization. Anal. Chem..

[6] Liu J (2010). Development, characterization, and application of paper spray ionization. Anal. Chem..

[7] Brown H, Oktem B, Windom A, Doroshenko V, Evans-Nguyen K (2016). Direct analysis in real time (DART) and a portable mass spectrometer for rapid identification of common and designer drugs on-site. Forensic. Chemistry..

[8] Gao L, Sugiarto A, Harper JD, Cooks RG, Ouyang Z (2008). Design and characterization of a multisource hand-held tandem mass spectrometer. Anal. Chem..

[9] Espy RD (2014). Paper spray and extraction spray mass spectrometry for the direct and simultaneous quantification of eight drugs of abuse in whole blood. Anal. Chem..

[10] Wiley JS, Shelley JT, Cooks RG (2013). Handheld low-temperature plasma probe for portable ‘point-and-shoot’ ambient ionization mass spectrometry. Anal. Chem..

[11] Hajslova J, Cajka T, Vaclavik L (2011). Challenging applications offered by direct analysis in real time (DART) in food-quality and safety analysis. Trends Anal. Chem..

[12] Ackerman LK, Noonan GO, Begley TH (2009). Assessing direct analysis in real-time-mass spectrometry (DART®-MS) for the rapid identification of additives in food packaging. Food Addit. Contam., Part A: Chem., Anal. Control, Exposure Risk Assess..

[13] Steiner RR, Larson RL (2009). Validation of the direct analysis in real time source for use in forensic drug screening. J. Forensic Sci..

[14] Fouyer K, Lavastre O, Rondeau D (2012). Direct monitoring of the role played by a stabilizer in a solid sample of polymer using direct analysis in real time mass spectrometry: The case of Irgafos 168 in polyethylene. Anal. Chem..

[15] Grange AH (2013). Semi‐quantitative analysis of contaminants in soils by direct analysis in real time (DART) mass spectrometry. Rapid Commun. Mass Spectrom..

[16] Wang LP, Zhao P, Zhang F, Li Y, Pan C (2012). Direct analysis in real time mass spectrometry for the rapid identification of four highly hazardous pesticides in agrochemicals. Rapid Commun. Mass Spectrom..

[17] Zhao Y, Lam M, Wu D, Mak R (2008). Quantification of small molecules in plasma with direct analysis in real time tandem mass spectrometry, without sample preparation and liquid chromatographic separation. Rapid Commun. Mass Spectrom..

[18] Zhou M, McDonald JF, Fernández FM (2010). Optimization of a direct analysis in real time/time-of-flight mass spectrometry method for rapid serum metabolomic fingerprinting. J. Am. Soc. Mass Spectrom..

[19] Yu S, Crawford E, Tice J, Musselman B, Wu JT (2009). Bioanalysis without sample cleanup or chromatography: the evaluation and initial implementation of direct analysis in real time ionization mass spectrometry for the quantification of drugs in biological matrixes. Anal. Chem..

[20] Curtis M (2010). Direct analysis in real time (DART) mass spectrometry of nucleotides and nucleosides: Elucidation of a novel fragment [C5H5O]+ and its in-source adducts. J. Am. Soc. Mass Spectrom..

[21] Vaclavik L, Zachariasova M, Hrbek V, Hajslova J (2010). Analysis of multiple mycotoxins in cereals under ambient conditions using direct analysis in real time (DART) ionization coupled to high resolution mass spectrometry. Talanta..

[22] Lesiak, A. D., Fowble, K. L. & Musah, R. A. A rapid, high-throughput validated method for the quantification of atropine in datura stramonium seeds using direct analysis in real time-high resolution mass spectrometry (DART-HRMS). *Methods in Molecualr Biology* 1810 *Chapter* 18. 207–215 (2018). 10.1007/978-1-4939-8579-1_18 29974430

[23] Habala L, Valentova J, Pechova I, Fuknova M, Devinsky F (2016). DART – LTQ ORBITRAP as an expedient tool for the identification of synthetic cannabinoids. Legal Medicine..

[24] Gomez-Rios GA, Gionfriddo E, Poole J, Pawliszyn J (2017). Ultrafast Screening and Quantitation of Pesticides in Food and Environmental Matrices by Solid-Phase Microextraction−Transmission Mode (SPME-TM) and Direct Analysis in Real Time (DART). Anal. Chem..

[25] Sisco E, Verkouteren J, Staymates J, Lawrence J (2017). Rapid detection of fentanyl, fentanyl analogues, and opioids for on-site or laboratory based drug seizure screening using thermal desorption DART-MS and ion mobility spectrometry. Forensic Chemistry..

[26] Saang’onyo D, Selby G, Smith DL (2012). Validation of a direct analyis in real time mass spectrometry (DART-MS) method of the quantitation of six carbon sugars in a saccharification matrix. Anal. Methods..

[27] Busman M, Bobell JR, Maragos CM (2015). Determination of the aflatoxin M1 (AFM1) from milk by direct analysis in real time-mass spectrometry (DART-MS). Food Control..

[28] Grange AH (2013). Semi-quantitative analysis of contaminants in soils by direct analysis in real time (DART) mass spectrometry. Rapid Commun. Mass Spectrom..

[29] Wang X (2014). Rapid quantification of highly polar trimethyl phosphate in wastewater via direct analysis in real-time mass spectrometry. J. Chromatogr. A..

[30] Nilles JM, Connell TR, Durst HD (2009). Quantitation of Chemical Warfare Agents Using the Direct Analysis in Real Time (DART) Technique. Anal. Chem..

[31] Maeno K, Shida Y, Shimada H (2017). Direct quantitative analysis of the natural moisturizing factor (NMF) in the stratum corneum by direct analysis in real time mass spectrometry (DART-MS). Anal. Methods..

[32] Horii I, Nakayama Y, Obata M, Tagami H (1989). Stratum corneum hydration and amino acid content in xerotic skin. Br. J. Der-matol..

[33] Spier H, Pascher G (1966). Free amino acids and water soluble peptides in stratum corneum and skin surface film in human beings. Hautarzt..

[34] Visscher M, Robinson M, Wickett RR (2010). Regional variation in the free amino acids in the stratum corneum. J. Cosmet. Sci..

[35] Robinson M, Visscher M, Laruffa A, Wickett R (2010). Natural moisturizing factors (NMF) in the stratum corneum (SC). I. Effects of lipid extraction and soaking. J. Cosmet. Sci..

[36] Robinson M, Visscher M, Laruffa A, Wickett R (2010). Natural moisturizing factors (NMF) in the stratum corneum (SC). II. Regeneration of NMF over time after soaking. J. Cosmet. Sci..

[37] Sylvie VS, Frédéric B (2007). Skin hydration: a review on its molecular mechanisms. J. Cosmet. Dermatol..

[38] Yamamoto-Tanaka M (2014). Mesotrypsin and caspase-14 participate in prosaposin processing. J. Biol. Chem..

[39] Sakabe J (2013). Kallikrein-related peptidase 5 functions in proteolytic processing of profilaggrin in cultured human keratinocytes. J. Biol. Chem..

[40] Hibino T (2013). Characterization of natural moisturizing factor (NMF)-generating enzymes and its relevance to barrier function. J. Soc. Cosmet. Chem. Jpn..

[41] Rawlings AV, Harding CR (2004). Moisturization and skin barrier function. Dermatol. Ther..

[42] Kamata Y (2009). Neutral cysteine protease bleomycin hydrolase is essential for the breakdown of deiminated filaggrin into amino acids. J. Biol. Chem..

[43] McGrath JA, Uitto J (2008). The filaggrin story: novel insights into skin-barrier function and disease. Trends. Mol. Med..

[44] Sandilands A (2009). Filaggrin in the frontline: role in skin barrier function and disease. J. Cell Sci..

[45] Wu C, Dill AL, Eberlin LS, Cooks RG, Ifa DR (2013). Mass spectrometry imaging under ambient conditions. Mass Spectrom. Rev..

[46] Bergman HM, Lundin E, Andersson M, Lanekoff I (2016). Quantitative mass spectrometry imaging of small-molecule neurotransmitters in rat brain tissue sections using nanospray desorption electrospray ionization. Analyst..

[47] Stauber J (2012). Quantitation by MS imaging: needs and challenges in pharmaceuticals. Bioanalysis..

[48] Annett U, Stefan H, Katrin K, Ulrich SS, Ferdinand VE (2016). Multigrid MALDI mass spectrometry imaging (mMALDI MSI). Anal. Bioanal. Chem..

[49] Muramoto S, Forbes TP, van Asten AC, Gillen G (2015). Test sample for the spatially resolved quantification of illicit drugs on fingerprints using imaging mass spectrometry. Anal. Chem..

[50] Luo Z (2018). Quantitative analysis of drug distribution by ambient mass spectrometry imaging method with signal extinction normalization strategy and inkjet-printing technology. Talanta..

[51] Voegeli R, Heiland J, Doppler S, Rawlings A, Schreier T (2007). Efficient and simple quantification of stratum corneum proteins on tape strippings by infrared densitometry. Skin Res. Tech..

[52] Sekimoto K (2014). Ionization characteristics of amino acids in direct analysis in real time mass spectrometry. Analyst..

[53] Badawy AAB, Morgan CJ, Turner JA (2008). Application of the Phenomenex EZ:faasttrade mark amino acid analysis kit for rapid gas-chromatographic determination of concentrations of plasma tryptophan and its brain uptake competitors. Amino Acids..

[54] Sugawara T, Kikuch K, Tagami H, Aiba S, Sakai S (2012). Decreased lactate and potassium levels in natural moisturizing factor from the stratum corneum of mild atopic dermatitis patients are involved with the reduced hydration state. J. Deratol. Sci..

[55] Badawy AAB (2012). The EZ:Faast family of amino acid analysis kits: application of the GC-FID kit for rapid determination of plasma tryptophan and other amino acids. Methods Mol. Biol..

[56] Ohta R, Inou M (2005). Composition of NMF in human stratum corneum, and its permeability and moisturizing behaviour. Bio Industry..

