# Supplementary material for: Direct Quantification of Natural Moisturizing Factors in Stratum Corneum using Direct Analysis in Real Time Mass Spectrometry with Inkjet-Printing Technique
Source: Sci Rep. 2019 Nov 28;9:17789. doi: 10.1038/s41598-019-54454-x (PMC6882842; doi:10.1038/s41598-019-54454-x)
Supplement: Supplementary file 1 — supplemental data [file 41598_2019_54454_MOESM1_ESM.pdf]

Direct Quantification of Natural Moisturizing Factors in Stratum Corneum using Direct Analysis in Real Time Mass Spectrometry with Inkjet-Printing Technique

Katsuyuki Maeno
Shiseido Global Innovation Center, 1-2-11, Takashima, Nishi-ku, Yokohama-shi, Kanagawa, 220-0011, Japan. Tel: +81-70-3859-1314; Fax: +81-45-222-1600; E-mail: katsuyuki.maeno@shiseido.com

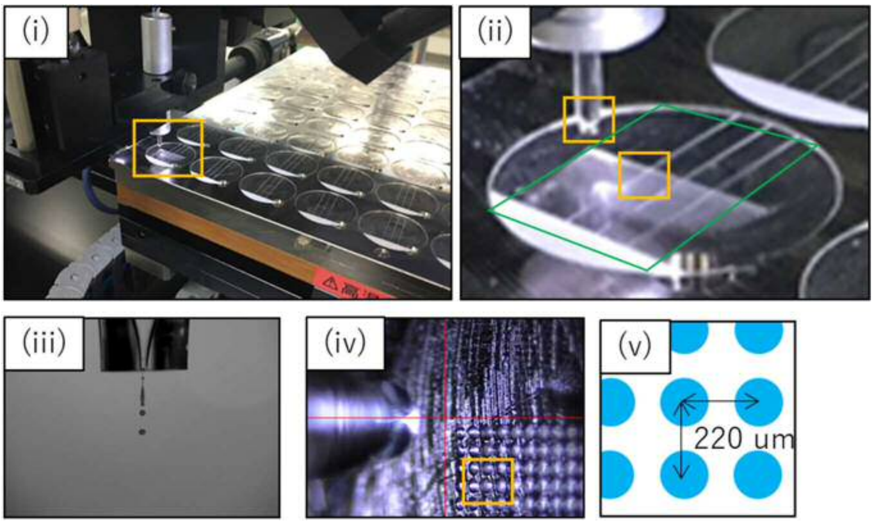

Figure S1 The SIL-IS mixture solution coating on each tape with LaboJet.

(i) A whole picture of LaboJet mainly composed of an inkjet head and a stage that can accommodate 81 (9 x 9) tapes on it. (ii) A zoomed in picture of the brown square area in (i). The SIL-IS mixture solution was coated inside the green square. (iii) A zoomed in picture of the upper brown square area in (ii). The inkjet head ejects a 1-nL droplet of the SIL-IS mixture solution. (iv) A zoomed in picture of the lower brown square area in (ii). A 1-nL droplet is placed regularly on the tape. (v) A schematic picture of the brown square area in (iv). Each droplet is designed to lie at an interval of 220 μm.

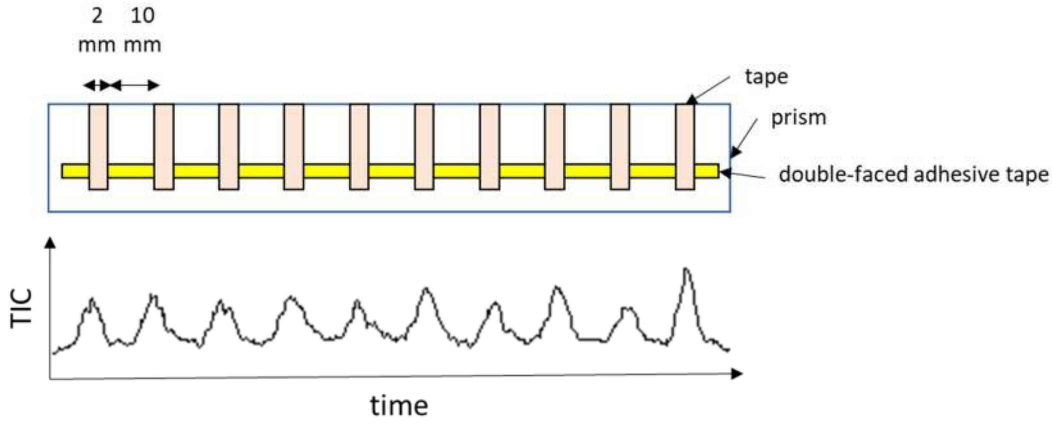

Figure S2 Top picture; a side view of the prism prepared in Fig. 1(d). Bottom picture: typical total ion current chromatogram when measuring 10 strips on the prism. Each peak corresponds to each tape.

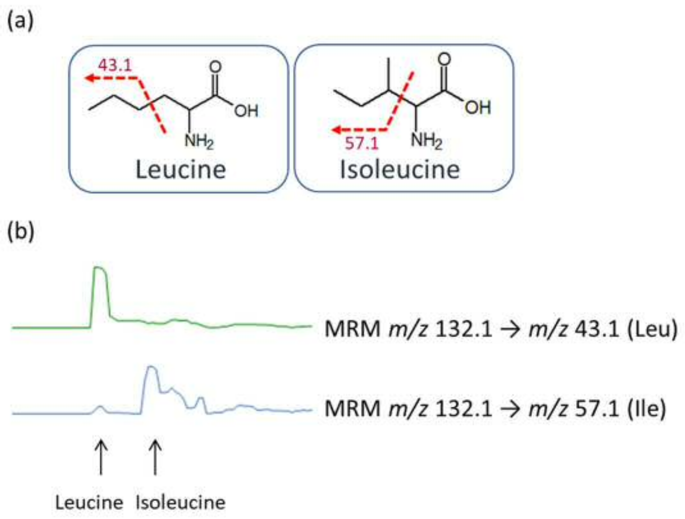

Figure S3 A specificity result of leucine and isoleucine. (a) Cleavage sites of leucine and isoleucine. (b) Measurement of leucine and isoleucine by MRM methods optimized for leucine and isoleucine.

Table S1 Comparison of two addition methods for the SIL-IS mixture solution

		Gly	Ala	Pro	Val	Thr	Met	Phe	Lys	Tyr	Ser
LaboJet	Area of NMF / Area of SIL-IS	1.2	1.1	1.2	1.1	1.2	1.2	1.2	1.2	1.1	0.92
	%RSD	6.3	15	8.9	5.8	4.3	7.7	7.1	3.2	23	23
Micropipetto	Area of NMF / Area of SIL-IS	0.9	0.77	0.94	0.92	0.9	0.82	0.95	2.3	2.5	2.3
	%RSD	22	24	25	21	22	24	20	100	89	98

Table S2 All the quantitative NMF values obtained by DART-MS and GC-MS; Unit: nmol of NMF / μg of Protein; S, Subject; L, Layer (For example, S1-L2 indicates the 2nd tape from the cheek of subject No.1)

<DART-MS>

	S1-L2	S1-L3	S1-L4	S2-L2	S2-L3	S2-L4	S3-L2	S3-L3	S3-L4	S4-L2	S4-L3	S4-L4	average
Val	2.49	3.39	4.49	1.37	1.87	2.28	1.23	1.98	1.47	1.12	1.45	1.93	2.09
Asn	1.56	2.36	2.63	0.91	1.09	1.15	0.97	1.47	1.21	0.94	1.04	1.46	1.40
Asp	2.09	2.62	4.17	1.41	2.49	2.32	1.91	2.56	2.33	2.05	2.56	3.71	2.52
Gln	4.41	6.82	12.88	1.78	3.71	4.48	2.07	4.61	2.70	2.14	2.80	5.45	4.49
Glu	3.73	5.08	6.60	1.70	3.88	3.76	2.50	4.17	2.89	2.43	3.96	4.27	3.75
Gly	4.97	9.41	11.07	1.77	3.42	7.36	2.49	3.41	4.61	5.03	6.41	11.82	5.98
His	2.08	2.14	2.48	0.56	2.59	2.91	1.11	2.00	1.53	3.16	1.73	3.29	2.13
Ile	1.05	1.25	1.68	0.32	0.55	0.67	0.47	0.72	0.48	0.40	0.38	0.65	0.72
Leu	1.96	1.69	2.25	0.18	0.15	0.14	0.57	0.76	0.50	0.12	0.02	0.30	0.72
Lys	2.27	4.41	6.16	0.96	2.15	2.58	1.19	3.81	2.03	0.77	1.79	2.23	2.53
Met	0.70	1.03	1.38	0.43	0.51	0.61	0.48	0.69	0.50	0.42	0.45	0.54	0.64
Phe	2.75	4.50	7.33	1.44	2.57	3.44	2.05	4.08	2.58	1.77	2.11	3.24	3.16
Pro	1.36	2.03	3.42	0.65	1.69	2.03	0.76	1.62	1.20	1.00	1.43	2.01	1.60
Ser	25.63	42.02	40.11	9.00	16.69	18.66	8.81	13.47	16.75	12.75	16.78	21.64	20.19
Thr	4.63	6.97	9.47	2.10	4.54	4.69	2.07	3.71	3.17	2.85	4.24	5.96	4.53
Trp	0.65	0.96	1.43	0.48	0.81	0.95	0.59	1.13	0.68	0.57	0.66	1.10	0.83
Tyr	2.42	3.47	4.80	1.00	2.38	3.06	1.43	3.30	2.13	1.65	2.44	3.06	2.59
Ala	2.90	12.39	6.82	2.19	3.53	10.72	3.22	3.71	2.54	3.21	7.06	6.00	5.36
Orn	1.24	2.10	4.68	0.89	3.24	2.44	0.63	2.09	1.59	1.24	2.74	3.68	2.21
PCA	8.97	8.31	9.57	15.04	12.17	6.83	5.11	7.77	5.59	4.45	5.88	4.45	7.85
Cit	3.77	16.10	8.87	2.84	4.59	13.94	4.18	4.83	3.30	4.18	9.18	7.80	6.97
UCA	50.28	43.08	93.75	33.01	28.68	26.51	24.44	38.80	24.40	27.18	35.28	29.30	37.89
Urea	10.35	18.70	17.49	10.51	3.30	4.20	2.80	3.13	2.48	2.97	1.70	0.72	6.53

<GC-MS>

	S1-L2	S1-L3	S1-L4	S2-L2	S2-L3	S2-L4	S3-L2	S3-L3	S3-L4	S4-L2	S4-L3	S4-L4
Val	1.41	1.59	2.28	0.82	0.95	0.90	1.00	0.83	1.06	0.93	1.15	1.33
Asn	0.61	0.90	1.30	0.37	0.40	0.31	0.42	0.40	0.39	0.27	0.41	0.52
Asp	2.45	2.74	3.67	1.38	1.32	1.33	1.78	1.60	1.52	1.56	2.32	2.40
Gln	0.67	0.47	0.66	0.85	0.61	0.81	0.85	0.60	0.87	0.60	0.77	0.54
Glu	2.46	2.33	2.99	1.13	1.41	1.28	1.33	1.14	1.30	1.07	3.27	1.77
Gly	4.14	5.60	8.23	3.08	3.51	3.33	2.64	3.65	2.62	3.58	5.31	5.59
His	4.07	5.06	7.31	2.48	2.38	1.07	1.97	1.07	0.24	0.42	0.76	0.87
Ile	0.93	1.03	1.51	0.53	0.57	0.53	0.68	0.51	0.68	0.50	0.63	0.79
Leu	1.80	1.83	2.33	0.99	0.99	0.81	1.31	0.95	1.01	0.88	0.93	1.14
Lys	0.85	0.67	1.02	0.39	0.56	0.39	0.35	0.28	0.09	0.18	0.25	0.33
Met	0.13	0.14	0.16	0.07	0.06	0.09	0.10	0.04	0.02	0.03	0.09	0.11
Phe	1.03	0.87	1.20	0.60	0.63	0.57	0.65	0.60	0.63	0.54	0.64	0.65
Pro	0.67	0.75	1.04	0.48	0.53	0.52	0.41	0.38	0.45	0.49	0.67	0.73
Ser	8.36	12.25	16.96	7.16	7.59	6.94	3.78	3.48	1.69	3.70	6.49	10.99
Thr	2.15	2.69	4.01	1.58	1.82	1.63	1.20	1.02	0.80	1.29	2.04	2.26
Trp	0.26	0.20	0.27	0.15	0.16	0.09	0.13	0.08	0.03	0.08	0.10	0.12
Tyr	1.04	0.96	1.37	0.69	0.70	0.58	0.72	0.53	0.38	0.56	0.70	0.72
Ala	2.23	2.84	3.92	1.67	2.02	1.90	1.40	1.30	1.51	1.82	2.60	2.70
